# The NF-κB p50:p50:HDAC-1 repressor complex orchestrates transcriptional inhibition of multiple pro-inflammatory genes

**DOI:** 10.1016/j.jhep.2010.03.025

**Published:** 2010-09

**Authors:** Ahmed M. Elsharkawy, Fiona Oakley, Feng Lin, Graham Packham, Derek A. Mann, Jelena Mann

**Affiliations:** 1 Liver Group, Institute of Cellular Medicine, Newcastle University, Newcastle Upon Tyne, UK; 2 Cancer Research UK Centre, Cancer Sciences Division, University of Southampton School of Medicine, Southampton General Hospital, Southampton, UK

**Keywords:** HDAC, histone acetyltransferase, MMP, matrix metalloproteinase, CCl_4_, carbon tetrachloride, ChIP, chromatin immunoprecipitation, MAP3K, MAP kinase kinase kinase, IκB, inhibitor of kappa B, p50 NF-κB, HDAC-1, Inflammation, MMP-13, GM-CSF, CXCL10, CCL2, ChIP, Microarray

## Abstract

**Background & Aims:**

The pro-inflammatory functions of NF-κB must be tightly regulated to prevent inappropriate tissue damage and remodelling caused by activated inflammatory and wound-healing cells. The p50 subunit of NF-κB is emerging as an important repressor of immune and inflammatory responses, but by mechanisms that are poorly defined. This study aims to delineate p50 target genes in activated hepatic stellate cells and to outline mechanisms utilised in their repression.

**Methods:**

Hepatic stellate cells were isolated from *nfkb1*(p50)-deficient or Wt mice and gene expression compared using microarray. Target genes were verified by qRT-PCR and p50-mediated HDAC-1 recruitment to the target genes demonstrated using chromatin immunoprecipitation.

**Results:**

We identify p50 as transcriptional repressor of multiple pro-inflammatory genes including *Ccl2*, *Cxcl10*, *Gm-csf*, and *Mmp-13*. These genes are over-expressed in *nfkb1*(p50)-deficient mice suffering from chronic hepatitis and in fibrogenic/inflammatory hepatic stellate cells isolated from *nfkb1*^−/−^ liver. We identify *Mmp-13* as a *bona-fide* target gene for p50 and demonstrate that p50 is required for recruitment of the transcriptional repressor histone deacetylase (HDAC)-1 to κB sites in the *Mmp-13* promoter. Chromatin immunoprecipitations identified binding of HDAC-1 to specific regulatory regions of the *Ccl2*, *Cxcl10*, *Gm-csf* genes that contain predicted κB binding motifs. Recruitment of HDAC-1 to these genes was not observed in *nfkb1*^−/−^ cells suggesting a requirement for p50 in a manner similar to that described for *Mmp-13*.

**Conclusions:**

Recruitment of HDAC-1 to inflammatory genes provides a widespread mechanism to explain the immunosuppressive properties of p50.

## Introduction

A limited but effective inflammatory response is essential for ensuring neutralisation of microbial pathogens following infection and for the removal of damaged and necrotic cells resulting from tissue trauma. However, this response is not without risk. If inflammation fails to resolve (as in chronic disease) then the tissue can be subject to significant cellular damage caused by highly toxic soluble factors (e.g. proteolytic enzymes) released by inflammatory cells (neutrophils, eosinophils and macrophages) that persist at the site of infection/trauma. For a “healthy” inflammatory response it is therefore important that the process follows its natural course from the initiation phase through to the resolution phase [16,33]. Where this course is perturbed, and inflammation fails to resolve, then there is potential for tissue damage and pathological states [1,35]. Tight regulation of the expression of inflammatory genes such as cytokines, chemokines, cell adhesion molecules, and proteolytic enzymes is a key factor in ensuring the appropriate induction and subsequent resolution of inflammation [16,33].

NF-κB is now firmly established as a cardinal transcriptional regulator of inflammatory gene expression and it is now emerging that different forms of NF-κB have distinct functions to play in the inflammatory response [13]. Mammalian NF-κB functions as homodimers or heterodimers generated from combinations of five Rel family proteins (RelA, c-Rel, RelB, p50 and p52). The five Rel family members share many conserved structural and functional features, for example they all have a similar Rel homology domain that enables dimerisation and DNA binding [12]. However, there are also distinctive structural and functional properties for each subunit which influence the activities of a particular NF-κB dimer. RelA and p50 are constituents of the so-called canonical NF-κB pathway that is thought to play a dominant role in the control of inflammation [28]. The canonical pathway is activated in response to a wide variety of inflammatory stimuli including cytokines (e.g. TNF-α and IL-1β) and ligands for the Toll-like receptors (e.g. lipopolysaccharide). RelA:p50 NF-κB dimers are target end-points for the signaling pathways triggered by these stimuli. Under resting conditions these dimers are associated with the inhibitory protein IκBα. IκBα lowers the efficiency of NF-κB transport to the nucleus and interferes with DNA binding [30]. Inflammatory signals culminate in the release of NF-κB from IκBα enabling interaction with target κB sequence motifs in the regulatory regions of genes controlling the inflammatory response. In general terms, RelA:p50 functions to increase the rate of transcription of inflammatory genes. One way in which resolution of inflammation is assured is that RelA:p50 stimulates transcription of IκBα to provide a rapid negative feedback mechanism. However, several additional regulatory checkpoints operate to ensure tight control of NF-κB-directed inflammation, including mechanisms provided by the unique properties of the p50 subunit [41,43].

The p50 subunit lacks a transactivation domain and as a consequence it can only stimulate transcription when either partnered with one of the transactivation domain-containing subunits RelA, c-Rel or RelB (p52 also lacks a transactivation domain), or when complexed with a transcriptional co-regulator such as Bcl3 or CBP. Homodimers of p50 can act as transcriptional repressors of pro-inflammatory genes and in this context are features of endotoxin-tolerant and tumour-associated macrophages [7,29,31,43]. Mice lacking the gene (*nfkb1*) encoding the p50 subunit have highly elevated inflammatory responses to insults of the lung, liver, and kidney suggesting that perhaps the predominant role of p50 is to operate as a negative regulator of NF-κB-directed inflammation [14,24,26].

We have previously reported that *nfkb1*^−/−^ mice suffer severe chronic inflammation and fibrosis in a model of iterative toxic injury of the liver [24]. An important cellular regulator of the hepatic inflammatory and fibrogenic response is the hepatic stellate cell. This is a non-parenchymal liver cell that in response to injury transdifferentiates into a wound-healing myofibroblasts [8,9]. In addition, recent data has shown that hepatic stellate cells act as antigen presenting cells within the liver [37,39]. These properties suggest a role for hepatic stellate cells as important regulators of hepatic inflammation. In our previous study we noted a perturbation of inflammatory signaling in *nfkb1*^−/−^ hepatic stellate cells [24]. For example, these cells aberrantly expressed TNF-α (both *in vivo* and *in vitro*) which could be attenuated by reconstitution of p50. We therefore concluded that *nfkb1*^−/−^ hepatic stellate cells are a useful model system to identify inflammatory genes that are subject to transcriptional repression by p50, and subsequently to determine how p50 inhibits transcription. In the present study, we identify 107 genes that are over-expressed in *nfkb1*^−/−^ hepatic stellate cells and by studying p50 regulation of 4 of these genes (*Mmp-13*, *Gm-csf*, *Ccl2*, and *Cxcl10*) we demonstrate that recruitment of the histone deacetylase (HDAC)-1 transcriptional repressor is a common mechanism by which p50 functions as a negative regulator of inflammation.

## Materials and methods

Animal studies have been reviewed and approved by the Local Ethical Committee and UK Home Office.

### Cell isolation and culture

Mouse hepatic stellate cells were isolated from C57Bl6 wild type or *nfkb1*^−/−^ livers as previously described [24] by incubation of liver segments with collagenase and pronase, followed by discontinuous density centrifugation in 12% Optiprep (Invitrogen). Hepatic stellate cells were cultured on plastic in Dulbecco’s modified Eagle’s medium, supplemented with 100 U/ml penicillin, 100 μg/ml streptomycin, 2 mM l-glutamine, and 16% fetal calf serum. Cell cultures were maintained at 37 °C at an atmosphere of 5% CO_2_.

### Microarray analysis

To perform the microarray experiment, hepatic stellate cells were isolated from groups of 3 animals from both C57Bl6 wild type and *nfkb1*^−/−^ mice as previously described [24]. The cells were activated on plastic and passaged. Cells were harvested between passages 4 and 7 for microarray analysis. Four independent preparations of wild type and *nfkb1*^−/−^ cells were used for the experiment. RNA was extracted using Qiagen RNAeasy Kit (Qiagen UK) according to the manufacturer’s instructions. RNA was only used if the A260/A280 ratio was between 1.8 and 2.1. Each RNA prep was used to prepare a probe that was hybridised to a single array. cRNA was synthesized from total RNA using the CodeLinkTM Expression Assay Reagent Kit (GE Healthcare, Amersham, UK) according to the manufacturer’s instructions. Probes were prepared by *in vitro* transcription, labelling, and fragmentation of the DNA and were hybridised to GE Healthcare CodeLinkTM Uniset 10K murine gene BioArrays (GE Healthcare, Amersham, UK) containing 10,458 probe sets. The arrays were washed according to the manufacturer’s instructions and results visualised with a GenePixTM 4100A microarray scanner (Molecular Devices, Wokingham, UK). Means of duplicates were used to analyse fold differences between wild type and *nfkb1*^−/−^ expression patterns and only genes with a greater than 2-fold difference and a *p* <0.05 (by Student’s *t*-test) were considered further. Ontological analysis of the genes differentially regulated between the two genotypes was performed using Genespring software.

### Chronic CCl_4_ liver injury model

Fibrogenesis was induced by 12-week CCl_4_ treatment of 6 week old *nfkb1*^−/−^ or age matched WT littermates [24]. Mice were injected IP twice weekly with CCl_4_/olive oil in a 1:3 [vol/vol] ratio at 1 μl/g body weight. Twenty-four hours after the final CCl_4_ administration, animals were sacrificed and liver samples prepared.

### RNA extraction, cDNA synthesis and quantitative reverse transcriptase-polymerase chain reaction (RT-PCR)

Total RNA was purified from isolated cells using the RNeasy purification kit (Qiagen, UK) following the manufacturer’s instructions and was used to generate cDNA utilising a random hexamer primer [p(dN)6] and MMLV reverse transcriptase (Promega, UK). Primers for mouse *Cxcl10*, *Mmp-13*, *Ccl2*, *Mmp3*, and fibromodulin were all purchased as Assays on demand (Applied Biosystems). All PCRs were normalized to the internal control (18S) and relative level of transcriptional difference calculated using the following equation: [1/(2*A*)] × 100.

### Crosslinked chromatin immunoprecipitation (XChIP) assay

Unless otherwise specified, ChIP assay was carried out using 20 μg crosslinked chromatin prepared from activated hepatic stellate cells [18]. Antibodies used for immunoprecipitation were raised against p50 (AbCam UK), p65 (Santa Cruz), and HDAC-1 (Upstate UK). Ten micrograms of each antibody or appropriate irrelevant antibody control was used in each chip reaction. chip primers used in the study were as follows – human MMP-13 promoter 5′-aca ttg agt ttt ggg tta ttg-3′ (sense) and 5′-ctt gat gac ttg gag gtg cta-3′ (anti-sense); mouse *Mmp-13* promoter 5′-gcc aga gaa aaa tga ttg agc-3′ (sense) and 5′-ccc tgg gga taa ggt cat ct-3′ (anti-sense); rat MMP-13 promoter 5′-ccc agt gaa gtg aaa aat-3′ (sense) and 5′-gca gtg cct gga gtc tct-3′ (anti-sense); mouse *Gm-csf* promoter κB4 site 5′-ctg ggg aga cag caa aga ag-3′ (sense) and 5′-gca ctt gag acc ctg aga gg-3′ (anti-sense); mouse *Gm-csf* promoter κB3 site 5′-cga ttc atc aga gct cac ca-3′ (sense) and 5′-ctg ggt ggc ttg tat gtc ct-3′ (anti-sense); mouse *Gm-csf* promoter κB2 site 5′-ttt gtc tct ggg tgg aaa cc-3′ (sense) and 5′-aaa ggc tcc att gca tca tc-3′ (anti-sense); mouse *Gm-csf* promoter κB1 site 5′-tcg aaa gcc ctc act tct gt-3′ (sense) and 5′-cat gtc aag gtg gag gag gt-3′ (anti-sense); mouse *Ccl2* promoter κB3 site 5′-ggc tgg gga ttg atg ttc ta-3′ (sense) and 5′-tgg aaa ttc cca ttc tga gg-3′ (anti-sense); mouse *Ccl2* κB2 site 5′-atg tga gag cgc cac tct tt-3′ (sense) and 5′-tgg tag ctc tct gcc ctg tt-3′ (anti-sense); mouse *Ccl2* κB1 site 5′-caa ggc ctg ata acc aag ga-3′ (sense) and 5′-ggg gaa aga ggg aag aat tg-3′ (anti-sense); mouse *Cxcl10* promoter κB3 site 5′-acc ata ggg agc gga ctc tt-3′ (sense) and 5′-ttg aaa gca gcc ctt tga ct-3′ (anti-sense); *Cxcl10* promoter κB2 site 5′-tca aag ggc tgc ttt caa gt-3′ (sense) and 5′-tcc aga ctt gcc tgt gtc tg-3′ (anti-sense); mouse *Cxcl*10 κB1 site 5′-tcc aag ttc atg ggt cac aa-3′ (sense) and 5′-gat gtc tct cag cgg tgg at-3′ (anti-sense). Each PCR was performed in triplicate and the analysis was repeated three times from independent ChIP experiments. Real-time PCR analysis was performed on an ABI 7500HT sequence detection system. In brief, qPCRs comprised of 20 ng of cDNA template, 15 pmol each of sense and anti-sense oligonucleotide primers, and 6.5 μl of Jumpstart SYBR green master mix (Sigma) in a total reaction volume of 13 μl. After the initial 20 s incubation at 94 °C, qPCRs were performed using a 20 s annealing at 55 °C followed by a 30 s elongation step at 72 °C and a 5 s denaturation step at 94 °C. After each run, a dissociation curve was performed to ensure that no primer dimers contaminated the quantification and that the product had the expected melting temperature. A signal intensity value for each sample was calculated from the average of the experiments. Average values of eluates were normalized to average values of inputs after subtracting the background control, and differences between samples calculated using the following equation: [1/(2*A*)] × 100. Detailed ChIP protocol is available on request.

### EMSA

NF-κB DNA binding was determined by EMSA as previously described [25] using a ^32^P end-labeled double-stranded oligonucleotide probe (Promega) containing a consensus NF-κB site. Nuclear extracts were prepared from p50 wt or EM1 and EM2 mutant transfected LX2 cells using a previously published protocol [25]. Harvested cells were washed twice in ice-cold phosphate-buffered saline prior to lysis in Buffer A supplemented with 0.2% Nonidet P-40, 0.5 mm 4-(2-aminoethyl)benzenesulfonyl fluoride, 0.2 mm EDTA, and 15 μg/ml aprotinin. Lysates were centrifuged for 10 s at 13,000 rpm to collect crude nuclear pellets. Supernatants were discarded and pellets were washed twice in lysis buffer without NP-40 prior to resuspension in Buffer C supplemented with 0.5 mm 4-(2-aminoethyl) benzenesulfonyl fluoride, 0.2 mm EDTA, and 15 μg/ml aprotinin. All buffers contained protease inhibitor cocktail (Sigma, UK). After a 10-min incubation on ice with occasional vortexing, the extracts were cleared of insoluble nuclear material by centrifugation at 13,000 rpm for 30 s. Cleared nuclear extracts were transferred to fresh Eppendorf tubes, and their protein content was determined using the Bradford DC assay kit (Bio-Rad). EMSA reactions were assembled on ice and consisted of an initial 10-min incubation of 4 μl of Buffer C containing 2–10 μg of nuclear protein extract and 12 μl of water containing 2 μg of poly(dI–dC). Four microlitres of water containing 0.4 ng of radiolabeled double-stranded probe was then added to the reaction and, after mixing, was incubated for a further 20 min. EMSA reaction mixtures were then resolved by electrophoresis on an 8% non-denaturing polyacrylamide gel (37:5:1).

### Transfections and reporter gene assays

LX2 and 3T3 cells were transfected by the non-liposomal Effectene protocol (Qiagen) according to the manufacturer’s instructions. Luciferase assays were performed using a dual luciferase kit (Promega) according to the manufacturer’s instructions. MMP-13 promoter-driven expression of firefly luciferase was normalized to protein concentration of the samples.

### Constructs

MMP-13 promoter-luciferase reporter constructs were a kind gift from Dr. Ernesto Canalis (St. Francis Hospital and Research Centre, Connecticut, USA). Studies described in this paper used a 721 bp fragment of MMP-13 promoter which encompasses −721 to +1 of rat MMP-13 promoter inclusive of NF-κB site at −498. The second MMP-13 promoter-luciferase reporter contains 227 bp fragment of MMP-13 promoter encompassing −227 to +1 of rat MMP-13 promoter.

### Generation of p50 mutants EM1 and EM2

p50 mutant constructs that are unable to homodimerise, namely EM1 and EM2, were generated using two-step recombinant PCR as described previously [19]. EM1 carries Y267A/L269A mutations, whereas EM2 has further two residues mutated so to make Y267A/L269A/F307A/V310A. Flag-tagged p50 wt expression construct was used as template with following mutation introducing primers – EM1 sense 5′-ga ggg gag gaa att gct ctt gct tgt gac aaa gtt c-3′ (mutated base pairs underlined in all primers) and EM1 anti-sense 5′-t ctg aac ttt gtc aca agc aag agc aat ttc ct-3′. Once generated, EM1 construct was used as template to generate EM2, utilising following mutation introducing primers – EM2 sense 5′-cat aga caa gcc gcc att gcc ttc aaa act cca-3′ and EM2 anti-sense 5′-tgg agt ttt gaa ggc aat ggc ggc ttg tct atg-3′. Both mutants were cloned into pcDNA3 using HindIII/XbaI restriction sites.

## Results

### Loss of nfkb1^−/−^ results in widespread changes of genes controlling inflammation and fibrosis

**Fig. 1:**
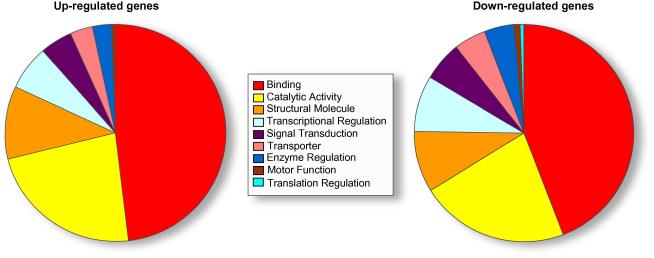
**Genespring analysis of the ontologies of genes that are up or down-regulated in*nfkb1*^−/−^ activated HSCs as measured by Amersham 10K murine microarray**.

**Fig. 2:**
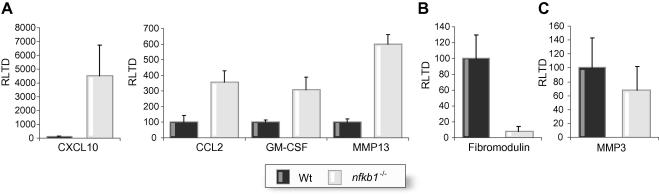
**Verification of differentially expressed genes in nfkb1^−/−^HSCs.** (A) CXCL10, CCL2, GM-CSF, and MMP-13 mRNA levels were quantified by qRT-PCR in wild type and *nfkb1*^−/−^ activated HSCs. Results are expressed as relative transcriptional difference as compared to the control ±SEM; *n* = 5. (B) Fibromodulin mRNA levels were quantified by qRT-PCR in wild type and *nfkb1*^−/−^ activated HSCs. Results are expressed as already stated ±SEM; *n* = 5. (C) MMP3 mRNA levels were quantified by qRT-PCR in wild type and *nfkb1*^−/−^ activated HSCs. Results are expressed as already stated ±SEM; *n* = 5.

**Fig. 3:**
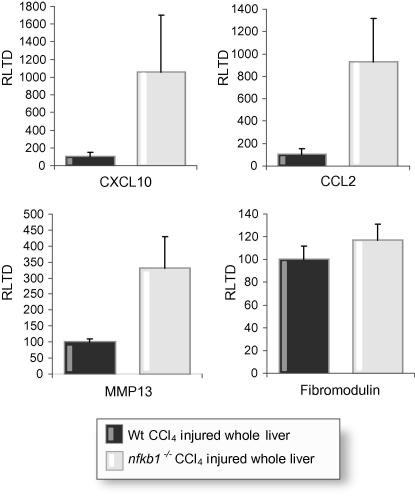
**Quantification of up-regulated gene targets in chronically CCl_4_-injured whole liver samples.** (A) CXCL10, CCL2, fibromodulin, and MMP-13 mRNA levels were quantified by qRT-PCR in wild type and *nfkb1*^−/−^ chronic CCl_4_-injured livers. Results are expressed as relative transcriptional difference as compared to the control ±SEM; *n* = 5.

Microarray and subsequent Genespring analysis revealed a total of 228 genes that are differentially regulated by 2-fold or greater between Wt and *nfkb1*^−/−^ hepatic stellate cells (Fig. 1 and Supplementary Tables 1 and 2). Of these genes, 107 were up-regulated and 121 were down-regulated and represented genes involved in a wide variety of physiological processes relevant to immunity and wound-healing (Fig 1). As *nfkb1*^−/−^ mice display increased susceptibility to carbon tetrachloride (CCl_4_)-induced inflammation and fibrosis [24], we were particularly interested in genes over-expressed in *nfkb1*^−/−^ hepatic stellate cells that regulate these processes. Over-expressed inflammatory/fibrogenic genes identified include MMP-13 (38-fold); IL-1β (35-fold); Arginase 2 (13-fold), which is required for synthesis of proline, a critical component of collagen; coagulation factor V (7-fold); IL-13 receptor α2 (7-fold), which mediates induction of TGF-β1 expression in response to IL-13; CXCL10 (7-fold); GM-CSF (6-fold); IL-11 (5-fold), a well known profibrogenic cytokine; the acute phase protein Ceruloplasmin (5-fold); Vanin 3 (4-fold), a pantetheinase implicated in inflammation; IL-15 (3-fold); and CCL2 (2.5-fold). To validate the microarray data we employed qRT-PCR to compare expression of MMP-13, CXCL10, GM-CSF, and CCL2 (up-regulated), fibromodulin (down-regulated), and MMP-3 (no difference) between Wt and *nfkb1*^−/−^ hepatic stellate cells. As shown in Fig. 2, qRT-PCR confirmed elevated expression of MMP-13, CXCL10, GM-CSF and CCL2, and reduced expression of fibromodulin in *nfkb1*^−/−^ hepatic stellate cells with again no difference observed for MMP-3. Differences in the fold-increases detected by microarray and qRT-PCR most likely reflect a variety of technical differentials between the two methodologies as well as differences in normalisation of microarray versus qRT-PCR data. We also confirmed that total hepatic expression of MMP-13, CXCL10, and CCL2 are elevated for chronic CCl_4_-injured *nfkb1*^−/−^ mice compared with Wt controls (Fig 3).

### MMP-13 gene transcription is suppressed by the p50 product of nfkb1

**Fig. 4:**
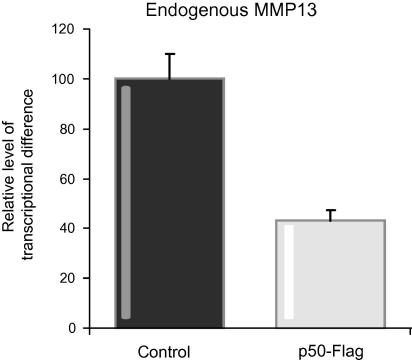
**Quantification of endogenous levels of MMP-13 in 3T3 cells transfected with p50.** Ten centimetres of dishes of 3T3 cells was transfected with 3 μg of p50-Flag expression vector or control. Cells were harvested 48 h later, RNA prepared and used in qRT-PCRs with primers specific for MMP-13; *n* = 4.

**Fig. 5:**
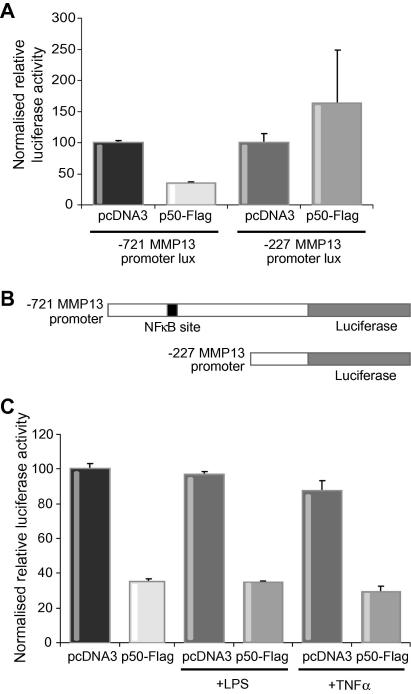
**p50 suppresses MMP-13 promoter activity.** (A) Human HSC cell line LX2 was transfected with 1 μg of −721 bp long (NF-κB binding site containing) or −227 bp short (no NF-κB site) MMP-13 promoter luciferase construct and co-transfected with 2 μg p50-Flag expression or control vector. Transfections were harvested 48 h later and luciferase assay carried out on cell lysates. Results are normalized to protein concentration; *n* = 3. (B) Schematic representation of MMP-13 promoter-luciferase reporters used in A and C. (C) Human HSC cell line LX2 was transfected with 1 μg of −721 bp long (NF-κB binding site containing) MMP-13 promoter luciferase construct, and co-transfected with 2 μg p50-Flag expression or control vector in triplicate. Each set of transfections was either treated with 10 ng/ml LPS (8 h prior to harvest) or TNF-α (4 h prior to harvest) or left untreated. Transfections were harvested 48 h after transfection and luciferase assay carried out on cell lysates. Results are normalized to protein concentration; *n* = 3.

Since MMP-13 was highly over-expressed in *nfkb1*^−/−^ hepatic stellate cells we were next interested to determine if the p50 product of *nfkb1*^−/−^ exerts any influence on MMP-13 expression and gene transcription. We initially showed that over-expression of p50 will suppress expression of endogenous MMP-13 (Fig. 4). To investigate MMP-13 transcription, the human hepatic stellate cell line LX2 [40] was transfected with MMP-13-promoter-luciferase reporter constructs (Fig. 5A) together with an expression vector for p50. Expression of p50 led to a 50% suppression of transcription from a luciferase reporter containing a 721 nucleotide long MMP-13 promoter. This effect was not observed with a shorter 227 nucleotide promoter containing only the proximal regulatory elements of the MMP-13 gene which by *in silico* analysis lacks a consensus κB site (Fig. 5B). Treatment with LPS or TNF-α failed to relieve p50-mediated suppression of transcription indicating the potential for p50 to inhibit MMP-13 expression even under highly pro-inflammatory conditions (Fig. 5C).

### HDAC-mediated repression of MMP-13 gene transcription

**Fig. 6:**
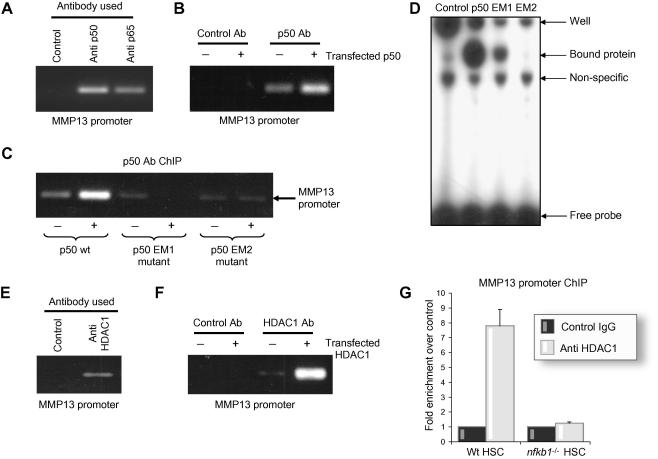
**p50 and HDAC-1 bind to MMP-13 promoter*in vivo*.** (A) Twenty micrograms of crosslinked chromatin obtained from rat myofibroblasts was incubated with 10 μg of anti-p50 or p65 antibody or non-specific control; ChIP assay was carried out and MMP-13 promoter amplified. (B) Human HSC cell line LX2 was transfected with 1 μg of p50-Flag expression vector, transfection harvested at 48 h, and crosslinked chromatin prepared. One hundred micrograms of obtained chromatin was incubated with 10 μg of anti-p50 antibody or non-specific control; ChIP assay was carried out and MMP-13 promoter amplified. (C) Human HSC cell line LX2 was transfected with 1 μg of p50-Flag, EM1 or EM2 expression vector, transfection harvested at 48 h and crosslinked chromatin prepared. One hundred micrograms of obtained chromatin was incubated with 10 μg of anti-p50 antibody or non-specific control; ChIP assay was carried out and MMP-13 promoter amplified. (D) Human HSC cell line LX2 was transfected with 1 μg of p50-Flag, EM1 or EM2 expression vector, transfection harvested at 48 h and nuclear extracts prepared. Five micrograms of nuclear extract was used in EMSA with NF-κB double-stranded γ-^32^P-labeled oligonucleotide probe. The gel shown is representative of two independent experiments. (E) Chromatin was prepared from human HSC cell line LX2. One hundred micrograms of obtained chromatin was incubated with 10 μg of anti-HDAC-1 antibody or non-specific control; ChIP assay was carried out and MMP-13 promoter amplified. (F) Human HSC cell line LX2 was transfected with 1 μg of HDAC-1 expression vector, transfection harvested at 48 h and crosslinked chromatin prepared. One hundred micrograms of obtained chromatin was incubated with 10 μg of anti-HDAC-1 antibody or non-specific control; ChIP assay was carried out and MMP-13 promoter amplified. (G) Crosslinked chromatin was prepared from Wt or *nfkb*^−/−^ HSCs. Twenty micrograms of chromatin was incubated with 10 μg of anti-HDAC-1 antibody or non-specific control; ChIP assay was carried out and MMP-13 promoter amplified.

**Fig. 7:**
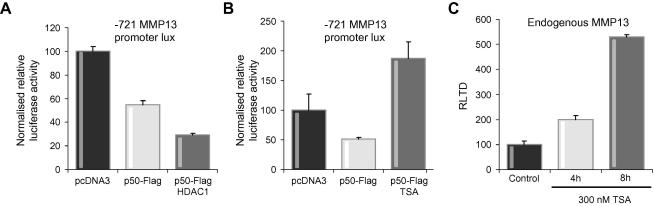
**Supressive effects of p50 on MMP-13 promoter are reversible using HDAC-1 inhibitor trichostatin A (TSA).** (A) Human HSC cell line LX2 was transfected with 0.5 μg of −721 bp long (NF-κB binding site containing) MMP-13 promoter luciferase construct and co-transfected with 1 μg p50-Flag expression/control vector along with 1 μg HDAC-1 expression/control vector. Transfections were harvested 48 h later and luciferase assay carried out on cell lysates. Results are normalized to protein concentration; *n* = 3. (B) Human HSC cell line LX2 was transfected with 0.5 μg of −721 bp long (NF-κB binding site containing) MMP-13 promoter luciferase construct and co-transfected with 1 μg p50-Flag expression/control vector. At 24 h, one-half of transfections containing p50 expression/control vector were treated with 300 nM TSA for 24 h. Cells were harvested at 48 h, luciferase assay carried out on cell lysates. Results are normalized to protein concentration; *n* = 3. (C) Wt mouse HSCs were treated with 300 nM TSA for 4 and 8 h, cells harvested and RNA prepared. MMP-13 mRNA levels were quantified by qRT-PCR, results are expressed as relative transcriptional difference as compared to the control ±SEM; *n* = 3.

NF-κB dimers regulate transcription by binding to κB sequences in the regulatory regions of target genes and via recruitment of co-activators (e.g. p300/CBP) or co-repressors (e.g. HDAC-1) which help to determine whether transcription is stimulated or suppressed, respectively [2,6,10,34,42]. The p50 subunit of NF-κB can form either pro-inflammatory heterodimers with RelA or anti-inflammatory p50:p50 homodimers. The latter are believed to actively suppress gene transcription through the recruitment of histone deacetylases including HDAC-1 [6,42]. ChIP analysis showed that both p50 and RelA are recruited to the MMP-13 promoter in hepatic stellate cells (Fig. 6A). Interaction of p50 with the MMP-13 promoter was confirmed with transfected p50 (Fig. 6B). This binding was, as expected, dependent on p50 dimerisation since mutant p50 proteins (EM1 and EM2), containing mutations that disrupt amino-acid residues in the Rel homology domain critical for p50 dimerisation, were not detected at the MMP-13 promoter by ChIP (Fig. 6B and C). EM1 expresses a mutant p50 that has amino acids Y270 and L272 mutated to alanine; these two amino-acid residues are predicted to influence homodimerisation and consequently DNA binding [32]. EM2 carries the same two mutations plus an additional two amino acid switches, F310 and V313 which are predicted to further perturb DNA binding of p50 [32]. These predictions were proved correct by EMSA analysis which showed reduced binding of EM1 to a consensus κB site compared with wild type p50 and undetectable binding of EM2 (Fig. 6D). We also detected binding of endogenous (Fig. 6E) and transfected HDAC-1 (Fig. 6F) to the MMP-13 promoter. Furthermore, recruitment of HDAC-1 to the MMP-13 promoter was dependent on p50 since we did not detect binding with ChIP in *nfkb1*^−/−^ cells (Fig. 6G). These data implicate HDAC-1 as a mediator of the suppressive effects of *nfkb1*/p50 on MMP-13 expression. To confirm this idea we determined the effects of a transfected HDAC-1 expression vector on MMP-13 promoter activity in LX2. As shown in Fig. 7A, transfection of p50 alone reduced MMP-13 transcription by 50% while co-transfection of p50 and HDAC-1 resulted in 70% suppression of transcription. By contrast treatment of hepatic stellate cells with the HDAC inhibitor trichostatin A prevented the repressive effects of transfected p50 and actually stimulated MMP-13 promoter activity above the control levels (Fig. 7B). Trichostatin A treatment also stimulated endogenous MMP-13 gene expression, with a 2-fold induction at 4 h treatment rising to 5-fold after 8 h. Taken together these data provide evidence that MMP-13 is a direct target for p50 which together with its co-repressor HDAC-1 acts to suppress MMP-13 transcription. As p50 is absent in *nfkb1*^−/−^ cells and *nfkb1*^−/−^ mouse liver, but there was no difference in HDAC-1 expression [24], we suggest that loss of this mechanism provides an explanation for the over-expression of MMP-13 in these cells and tissues.

### HDAC-1 recruitment is observed at multiple p50-repressed promoters

**Fig. 8:**
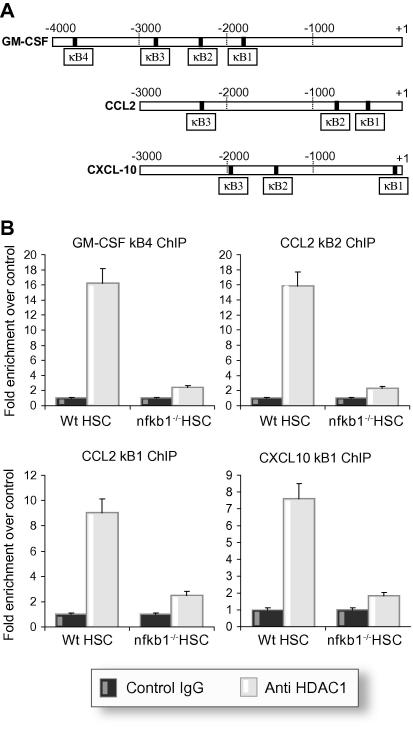
**HDAC-1 binds to GM-CSF, CCL2, and CXCL10 NF-κB sites*in vivo*.** (A) Schematic representation of putative NF-κB site within regulatory regions of GM-CSF, CCL2, and CXCL10 genes. (B) Crosslinked chromatin was prepared from Wt or *nfkb*^−/−^ HSCs. Twenty micrograms of chromatin was incubated with 10 μg of anti-HDAC-1 antibody or non-specific control; ChIP assay was carried out and regions surrounding putative NF-κB sites within GM-CSF promoter (B), CCL2 promoter (C), and CXCL10 promoter (D) were amplified. Several sites were found to bind HDAC-1 which are shown in the figure. Error bars represent ±SEM; *n* = 3.

We were next interested to determine if the elevated expression of GM-CSF, CCL2, and CXCL10 in *nfkb1*^−/−^ cells and liver was associated with p50-dependent recruitment of HDAC-1 to the regulatory regions of these genes. *In silico* analysis revealed multiple κB sites in the upstream promoter regions of the three genes (Fig. 8A). This information was used to select ChIP primers spanning two different κB per gene promoter. ChIP analysis of HDAC-1 recruitment to these regulatory sites was then determined in wild type and *nfkb1*^−/−^ cells (Fig. 8B). HDAC-1 binding was detected at one site in the GM-CSF gene (−3700), two sites in the CCl2 gene (−420 and −700) and at one site in the CXCL10 gene (−100). All of these HDAC-1 interactions were at near undetectable levels in *nfkb1*^−/−^ cells. The p50:HDAC-1 complex is therefore a transcriptional suppressor of multiple genes implicated in inflammation and tissue remodelling.

## Discussion

Inflammation is a highly regulated process that, if unchecked, can lead to conditions of chronic tissue damage and accumulation of fibrotic tissue. NF-κB is a cardinal regulator of inflammation and therefore continues to be extensively investigated with the hope of development of improved anti-inflammatory drugs [5,15]. Homodimers of the p50 subunit of NF-κB are features of the resolving phase of inflammatory responses and may operate as important transcriptional inhibitors of NF-κB-regulated inflammatory genes [4]. The physiological consequences of loss of p50 expression have been documented in *nfkb1*-deficient mice which display enhanced M1-driven inflammation characterised by perturbed expression of TNF-α, IFN-β, CXCL10, and iNOS [29,31]. These mice are also susceptible to severe inflammatory responses upon challenge of multiple organs [23,24,26]. Intratracheal infection of mice with *Escherichia coli* is associated with induction of RelA/p50 dimers in the lung at 3 h which is followed by appearance of p50/p50 dimers after 6 h, indicating a role for p50 homodimers in resolution of lung inflammation [22]. Challenge of *nfkb1*^−/−^ mice in this manner resulted in increased mortality, elevated neutrophil recruitment to the lung, and over-expression of KC, MIP-2, TNF-α, IL-6, and IL-1β at 6 h after infection [22]. In a model of glomerular nephritis, Panzer et al. reported a similar shift from RelA/p50 to p50/p50 dimers during transition from the initiation to resolution phase of kidney inflammation [26]. Mice lacking *nfkb1* displayed higher and prolonged expression of CCL2, CCL5, and TNF-α, increased numbers of infiltrating CD3^+^ cells and higher rates of mortality. We have previously reported more severe inflammation and fibrosis in *nfkb1*-deficient mice in a model of chronic liver fibrosis [24]. Taken together these models suggest that absence of p50 leads to the inappropriate expression of a variety of pro-inflammatory mediators resulting in severe unresolved inflammation. The present study improves our understanding of the role of p50 as a suppressor of inflammation by revealing that its recruitment of HDAC-1 provides a mechanism for the negative transcriptional regulation of at least four important pro-inflammatory genes (MMP-13, GM-CSF, CCL2, and CXCL10) that are over-expressed in the injured liver of *nfkb1*^−/−^ mice.cDNA microarray analysis revealed MMP-13 as the gene up-regulated by the greatest extent (37.7-fold) in *nfkb1*^−/−^ cells. MMP-13 is a protease with high selectivity for insoluble fibrillar collagens (in particular type I collagen) that is expressed in response to a variety of inflammatory mediators and in an NF-κB-dependent way [3,17,20,21]. The over-expression of MMP-13, in the context of the elevated fibrosis observed in chronic injured livers of *nfkb1*^−/−^ mice, may therefore seem somewhat paradoxical. However, Uchinami et al. have previously described the attenuation of liver fibrosis in MMP-13 knockout mice [36]. This effect results from MMP-13 being required for the initial inflammatory response to injury which is a major determinant of the subsequent fibrogenic response. MMP-13 probably stimulates inflammation by promoting the release of soluble active forms of inflammatory mediators (e.g. TNF-α) from their insoluble ECM-bound states [36]. Of note, Uchinami observed a dramatic reduction in neutrophil infiltration in the sinusoids of injured MMP-13-deficient mice [36]. A feature of injured *nfkb1*^−/−^ liver is extremely high numbers of infiltrating neutrophils [24]. Suppression of MMP-13 expression by p50/p50 may therefore be an important mechanism for limiting neutrophilic inflammation and tissue damage. As demonstrated here, transfection of p50 inhibits expression of endogenous MMP-13 and suppresses activity of an MMP-13 promoter-luciferase reporter. Furthermore, both endogenous and transfected p50 can be detected at the MMP-13 promoter by ChIP. Specificity of this binding was confirmed by showing that dimerisation- and DNA-binding-defective p50 proteins fail to ChIP to the promoter. Our data, however, does not exclude the possibility that NF-κB subunits other than p50 and RelA may bind selected target genes. MMP-13 is therefore a *bona-fide* target gene for p50 which under conditions of excess relative to RelA will promote repression of MMP-13 gene transcription. Such conditions are described in the resolving phase of inflammation where p50/p50 dominates over RelA/p50 dimers [11]. We conclude that absence of p50 is responsible for the highly elevated expression of MMP-13 (as well as CXCL10 and CCL2) in *nfkb1*^−/−^ cells and liver. This also supports the hypothesis that absence of p50 is responsible for the severe inflammatory responses of *nfkb1*^−/−^ mice. Of note, cells lacking the *nfkb1* gene effectively lack two proteins (p105 and p50) and also display defects in the expression and activity of the MAP3K Tpl2/Cot [27]. Hence, attributing phenotypes observed in *nfkb1*^−/−^ cells and mice to functions of p50 requires substantiation with additional experiments such as those we describe above.

As p50 lacks a transactivation domain its influences on transcription are dependent either upon dimerisation with RelA, c-Rel or RelB, or on the activities of co-regulators that assemble functional complexes with p50 at gene promoters. Co-regulators implicated as adapters of p50 function include HDAC-1, the IκB family protein Bcl3, and the histone acetylase CBP [10,34,42]. The nature and type of p50:co-regulator complex assembled, appear to be context dependent and may result in stimulation or repression of transcription. For example, p50:p50:HDAC-1 assembles at the TNF-α promoter to inhibit transcription, by contrast p50:p50:CBP assembles at the IL-10 promoter in macrophages responding to LPS and stimulates transcription [4]. The p50:p50:HDAC-1 prevents inappropriate transcription of TNF-α in fibrogenic hepatic stellate cells [24]. We were therefore interested to determine if a similar mechanism operates at the MMP-13 gene. Both endogenous and transfected HDAC-1 were associated with the MMP-13 promoter in wild type but not in *nfkb1*^−/−^ cells. HDAC-1 over-expression enhanced the repressive effects of p50 on the MMP-13 promoter, while treatment of cells with the HDAC inhibitor trichostatin A blocked the repressive effects of transfected p50 on MMP-13. Recruitment of a p50 and HDAC-1 containing complex at the TNF-α and MMP-13 genes raises the possibility that this complex operates as an orchestrator of the inhibition of multiple pro-inflammatory genes. We showed that HDAC-1 associated with κB site-containing upstream regions of genes encoding GM-CSF, CCL2, and CXCL10, all of which are expressed at higher levels in *nfkb1*^−/−^ cells and liver tissue compared with wild type. The interaction of HDAC-1 with these genes was detected in wild type but not in *nfkb1*^−/−^ cells confirming a requirement of p50 for recruitment of HDAC-1.

Previous work has identified the TNF-α, IL-6, and iNOS genes as being repressed by HDAC-1 in a p50-dependent manner [24,42]. A similar mechanism operates at the HIV-1 long terminal repeat to promote latency of the virus, with potential for pro-inflammatory signals to relieve this mechanism and stimulate viral replication [38]. By us now showing that p50-dependent recruitment of HDAC-1 orchestrates repression of MMP-13, GM-CSF, CCL2, and CXCL10 in a model of chronic liver inflammation and fibrosis, it can be concluded that the p50:p50:HDAC-1 complex is a master negative regulator of inflammation. The challenge is to define the molecular control of the recruitment and assembly of p50:p50:HDAC-1 and how this control is modulated in chronic unresolved states of inflammation.

## Conflict of Interest

The authors who have taken part in this study declared that they do not have anything to disclose regarding funding or conflict of interest with respect to this manuscript.

## Supplementary data

Supplementary data 1 List of down-regulated genes in p50^−/−^ mouse hepatic stellate cells as compared to the wt.

Supplementary data 2 List of up-regulated genes in p50^−/−^ mouse hepatic stellate cells as compared to the wt.
